# Does non-smoker identity following quitting predict long-term abstinence? Evidence from a population survey in England

**DOI:** 10.1016/j.addbeh.2015.01.026

**Published:** 2015-06

**Authors:** Ildiko Tombor, Lion Shahab, Jamie Brown, Caitlin Notley, Robert West

**Affiliations:** a Cancer Research UK Health Behaviour Research Centre, University College London, WC1E 6BT, UK; b School of Medicine Health Policy and Practice, University of East Anglia, NR4 7TJ, UK

**Keywords:** Smoking cessation, Smoker identity, Ex-smokers, Smoking Toolkit Study, Representative sample

## Abstract

**Aims:**

‘Categorical self-labels’ (e.g. thinking of oneself as a smoker or non-smoker) are important aspects of identity that can have a fundamental influence on behaviour. To explore the role identity aspects relating to smoking can play in smoking cessation and relapse, this study assessed the prospective associations between taking on a non-smoker identity following quitting and long-term abstinence.

**Methods:**

A representative sample of 574 ex-smokers in England who quit smoking in the past year was followed-up at three (*N* = 179) and six months (*N* = 163). Post-quit identity relating to smoking (‘I still think of myself as a smoker’ or ‘I think of myself as a non-smoker’), and demographic and smoking-related characteristics were assessed at baseline. Self-reported smoking abstinence was assessed at follow-ups.

**Results:**

Non-smoker identity was reported by 80.3% (95%CI 76.8–83.4) of recent ex-smokers. Younger age (*p* = 0.017) and longer abstinence (*p* < 0.001) were independently associated with a post-quit non-smoker identity. After adjusting for covariates, non-smoker identity (*p* = 0.032) and length of abstinence at baseline (*p* < 0.001) were associated with continued abstinence at three month follow-up, and baseline length of abstinence (*p* = 0.003) predicted continued abstinence at six months.

**Conclusions:**

The majority of people who quit smoking recently consider themselves as non-smokers. Younger people and those who have been abstinent for longer are more likely to take on a non-smoker identity. Ex-smokers who make this mental transition following a quit attempt appear more likely to remain abstinent in the medium term than those who still think of themselves as smokers.

## Highlights

• Smoker identities have been proposed to influence the success of quit attempts.
• Prospective associations were assessed in a representative sample of ex-smokers.
• The majority of people who quit in the past year consider themselves as non-smokers.
• Younger age and longer abstinence associated with a post-quit non-smoker identity.
• Adopting a non-smoker identity after quitting can predict medium-term abstinence.

## 1 Introduction

The proportion of ex-smokers who remain abstinent permanently is low (West, 2006). Some forms of pharmacological treatments can be effective in preventing relapse (Agboola, Mcneill, Coleman, & Leonardi-Bee, 2010; Hajek, Stead, West, Jarvis, & Lancaster, 2009), but systematic reviews have consistently found insufficient evidence of the effectiveness of behavioural relapse prevention interventions (Agboola et al., 2010; Hajek et al., 2009). To improve behavioural approaches that aim to help people sustain their initially successful quit attempts, we need to advance knowledge of factors that can contribute to long-term behaviour change. ‘Identity’ has a pivotal influence on health behaviours (Kearney & O'Sullivan, 2003; Oyserman, Fryberg, & Yoder, 2007; Vignoles, 2011; West & Brown, 2013) and it has been recognized as a potentially useful target for behaviour change interventions (Oyserman & Destin, 2010; Vignoles, 2011; West & Brown, 2013). Yet, there has been little published research on the role it can play in relapse or maintained abstinence in recent ex-smokers.

Approximately 75% of aided quit attempts (Ferguson, Bauld, Chesterman, & Judge, 2005) and 95% of unaided quit attempts fail within a year (Hughes, Keely, & Naud, 2004) with relapse being highest early on, typically in the first few weeks of abstinence (Hughes et al., 2004). Although the risk of relapse decreases sharply over time, it can remain substantial even after years of abstinence (Hawkins, Hollingworth, & Campbell, 2010; Hughes, Peters, & Naud, 2008; Yudkin et al., 2003). The process of relapse always starts with an initial lapse that involves a momentary suspension of inhibition to act on impulses to smoke triggered by external or internal stimuli (West & Brown, 2013), such as availability of cigarettes (Minami, Tran, & McCarthy, 2014) and negative mood (Vangeli, Stapleton, & West, 2010b), which can progress to complete relapse (Shiffman et al., 2006). Therefore, to achieve sustained behaviour change people need to exercise self-control to inhibit impulses to smoke and govern behaviour in accordance with desires not to smoke, and for which identity has been proposed to provide a potentially powerful motivational source (West & Brown, 2013).

Building primarily on the PRIME theory of motivation (West & Brown, 2013), we consider identity as a psychological construct that comprises people's mental representations of themselves, including their thoughts and images about themselves as they are at present and as they aspire to become in the future. As proposed by the social identity theory (Tajfel & Turner, 1986) and the self-categorisation theory (Turner, Hogg, Oakes, Reicher, & Wetherell, 1987), the PRIME theory also recognizes that important aspects of identity are ‘self-labels’ that describe the categories to which people consider that they belong (e.g. thinking of oneself as a smoker or non-smoker). In addition, people set ‘personal rules’ for themselves that specify a range of purposeful behaviours that they do or do not do as per their valued identity aspects (West & Brown, 2013). People are motivated to act in accordance with these identities, and if triggered by the context, salient identities can evoke identity congruent cognitions and behaviour (Oyserman & Destin, 2010; Oyserman et al., 2007). In the context of smoking cessation, making a deep identity change from being a smoker to a non-smoker after a quit attempt is more likely to prompt the formation of strong personal non-smoking rules and generate motives to adhere to this rule in any relevant moment when opposing motives arise (Lei Hum, Bulgiba, Shahab, Vangeli, & West, 2013; West & Brown, 2013).

Only a few studies have been published specifically about ex-smokers' identities following quitting. It has been reported that most people made the mental transition from ‘being a smoker’ to ‘being a non-smoker’ following quitting (Johnson et al., 2003; Vangeli, Stapleton, & West, 2010a; Vangeli & West, 2012); nevertheless, approximately a fifth of people who had quit smoking more than two years ago still identified themselves either with ‘reluctant non-smoker’ or ‘smoker who is not smoking’ self-labels (Vangeli et al., 2010a). Those who had been smoking for longer prior to their most recent quit attempt were more likely to retain a smoker identity despite stopping (Vangeli et al., 2010a). Both qualitative and quantitative evidence have suggested that failing to identify oneself with a firm non-smoker identity following quitting is associated with ex-smokers feeling vulnerable to future relapse (Johnson et al., 2003; Vangeli et al., 2010a).

Overall, research suggests that a non-smoker identity to which a person is committed could provide the basis for effective self-control and prevent people from acting on impulses and motivational forces that drive relapse by adhering to personal non-smoking rules. Therefore, this study assessed the prospective associations between taking on a non-smoker identity following a quit attempt and long-term abstinence in people who quit smoking in the past year. The study addressed the following questions:1.What is the proportion of recent ex-smokers in a nationally representative sample who report a non-smoker identity following a quit attempt?2.What socio-demographic and smoking-related characteristics are associated with having a post-quit non-smoker identity?3.What is the predictive relationship between post-quit non-smoker identity and long-term smoking abstinence?

## 2 Methods

### 2.1 Study design

This study used data that were collected in the Smoking Toolkit Study (STS) between April 2007 and February 2009. The STS involves an ongoing series of monthly household surveys to monitor smoking and smoking cessation figures and behaviour in nationally representative samples of adults age 16 and over in England (http://www.smokinginengland.info). Each month a new sample of approximately 1800 people (one adult member of each selected household) completes a face-to-face computer assisted baseline interview with a trained interviewer. All smokers and recent ex-smokers who quit in the past year were asked to provide consent to be re-contacted, and those who agreed then received postal follow-up questionnaires at three months from baseline, and again at six months if they returned the first. A detailed description of the survey methodology, including the sampling technique used, is reported elsewhere (Fidler et al., 2011). Ethical approval for the Smoking Toolkit Study was obtained from the University College London ethics committee.

### 2.2 Participants

The baseline sample comprised a representative sample of adult (age 16 and over) recent ex-smokers (*N* = 574) who reported having stopped smoking completely in the last year and provided data for all variables included in the current study. Of these, 179 (31.2%) and 163 (28.4%) completed the three month and six month follow-up questionnaires respectively.

### 2.3 Measures

Participants' demographic characteristics (gender, age and social grade) were collected. Social grade was measured according to the British National Readership Survey classification system and dichotomised into ABC1 (those with higher and intermediate professional/managerial, supervisory, clerical, junior managerial/administrative/professional occupations) and C2DE (those with skilled, semi-skilled and unskilled manual, and lowest grade occupations, or unemployed).

At baseline, post-quit identity relating to smoking was assessed by asking participants about the categorical self labels applied to themselves: ‘Which one of the following best describes you?’—‘I still think of myself as a smoker’ or ‘I think of myself as a non-smoker’. Data on the numbers of cigarettes per day (‘How many cigarettes per day did you usually smoke?’) and serious quit attempts in the past year were collected (‘How many serious quit attempts to stop smoking have you made in the last 12 months? By serious attempt I mean you decided that you would try to make sure you never smoked again. Please include any attempt that you are currently making’). To assess length of abstinence, participants were asked: ‘How long ago did your most recent serious quit attempt start? By most recent, we mean the last time you tried to quit’ (‘In the last week’; ‘More than a week and up to a month’; ‘More than 1 month and up to 2 months’; ‘More than 2 months and up to 3 months’; ‘More than 3 months and up to 6 months’; ‘More than 6 months and up to a year’). They were then further asked about the support they used in their most recent quit attempt (‘Which, if any, of the following did you try to help you stop smoking during the most recent serious quit attempt?’—‘Nicotine replacement product (e.g. patches/gum/inhaler) without a prescription’; ‘Nicotine replacement product on prescription or given to you by a health professional’; ‘Zyban (bupropion)’; ‘Champix (varenicline)’; ‘Attended an NHS Stop Smoking Service group’; ‘Attended an NHS Stop Smoking Service one to one counselling session’). In the analysis we dichotomised these into ‘unaided’ (did not use any quit aids) and ‘supported’ (used any of these listed quit aids).

At three month and six month follow-ups, participants were asked whether they smoke cigarettes (including hand-rolled cigarettes) at all nowadays (‘Yes’ or ‘No’).

### 2.4 Analysis

We calculated the prevalence of a non-smoker identity following a quit attempt by using weighted data to match English census data on age, gender and social grade. Logistic regression analyses were performed to assess the cross-sectional associations between post-quit non-smoker identity and baseline demographic and smoking-related characteristics, and to assess the prospective associations between post-quit non-smoker identity at baseline and smoking status at three month and six month follow-ups. To compare baseline characteristics of those who completed the follow-up questionnaires and those who were not followed-up at three months and six months, Pearson's *χ*^2^ and *t*-tests were used for categorical and continuous variables, respectively.

## 3 Results

Table 1 shows participants' characteristics at baseline, and of those who were followed-up and who were lost to follow-up at three months and six months respectively. Participants who completed the postal questionnaire at three months were slightly older than those lost to follow-up. Similarly, participants followed-up at six months were older, smoked more before quitting and were less likely to quit unaided.

In a representative sample of adult recent ex-smokers, 80.3% (95%CI 76.8–83.4) reported a non-smoker identity following stopping smoking completely in the past year. Table 2 shows that in univariable regression analyses, being younger, having been abstinent for longer and having quit unaided were significantly associated with reporting a non-smoker identity following quitting. After adjusting for all study variables, younger age and increased length of abstinence were associated with a non-smoker identity.

Table 3 reports the prospective logistic regression analyses that showed that males and those who reported a non-smoker identity and had been abstinent for longer at baseline were more likely to be abstinent at three month follow-up. Non-smoker identity and increased length of abstinence remained significant predictors after adjusting for all covariates. At six months, increased length of abstinence was the only significant predictor of smoking abstinence both in the univariable and multivariable logistic regressions.

## 4 Discussion

The majority—approximately 80%—of recent ex-smokers who quit smoking in the past year endorsed the statement ‘I think of myself as a non-smoker’ in a representative sample of adults in England. Younger age and increased length of abstinence were important contributors to a post-quit non-smoker identity, and taking on this identity following a quit attempt was associated with smoking abstinence in the medium-term over and above other predictors, including baseline length of abstinence.

Our prevalence estimate for the proportion of recent ex-smokers who used the ‘non-smoker’ self-label to describe themselves was considerably higher (80.3%) than previously reported in a study of a non-representative sample of treatment-seeking ex-smokers (Vangeli et al., 2010a). This study found that 46.9% of participants thought of themselves as a non-smoker after quitting in the past year; however, it is difficult to compare these findings due to the difference between the samples involved. In agreement with our results, this study also found that increased length of abstinence was significantly associated with the establishment of a non-smoker identity following quitting (Vangeli et al., 2010a). Consistent with previous work which found that younger people were more likely to feel bad about their ‘smoker’ self-label than older people (Tombor, Shahab, Brown, & West, 2013), younger people were more willing to take on a non-smoker identity after a quit attempt.

In terms of prospective predictors of long-term quit outcomes, previous studies have consistently found that baseline length of abstinence is one of the most important independent predictors of relapse (Hawkins et al., 2010; Wetter et al., 2004). In line with these findings, we found that those who had been abstinent for longer at baseline were less likely to report relapse at three month and six-month follow-ups. As it has been proposed by theories (Kearney & O'Sullivan, 2003; Oyserman et al., 2007; Vignoles, 2011; West & Brown, 2013), our findings showed that identifying oneself with a ‘non-smoker’ self-label could contribute to long-term behaviour change; however, the potentially protective influence of a post-quit non-smoker identity to avoid relapse was shown to be limited to the initial period after quitting. A plausible explanation for this could be that failing to establish a firm non-smoker identity, with positive feelings attached to it and a strong desire to become someone for whom smoking is not an option, may reflect underlying conflicts about stopping smoking (Wee, Shahab, Bulgiba, & West, 2011), and this could deter people from sticking to an absolute non-smoking rule.

Improving the effectiveness of behavioural treatments for relapse prevention would have huge public health benefits, and it could be particularly important for people, such as pregnant women, who cannot or do not want to use pharmacotherapy to support their quit attempts. This study cannot establish how far the label per se or factors that may lead to that label influence relapse risk. This is something that will not be easy to disentangle and will probably require an experimental study. However, this study provides at least a prima facie case that the label and its cognitive and emotional associations may play a role. Therefore, findings from this study suggest that harnessing identity change (towards a non-smoker identity) as part of behavioural smoking cessation support might improve the lasting success of quit attempts, and future research should explore the set of behaviour change techniques that can be effective to help people achieve this by incorporating a non-smoker self-label into a core part of their identity. In addition, further research is needed to replicate these findings in larger samples to assess if the prospective relationship between post-quit non-smoker identity and quit success can be found beyond a medium-term follow-up, and to explore if the pattern of results holds in special populations. We also need to better understand the contributing factors to a non-smoker identity following quitting smoking, and explore potential moderator and mediator effects between smoker identity and other predictors of relapse, such as urges to smoke (Herd, Borland, & Hyland, 2009).

One limitation of this study is that assessment of smoking abstinence did not involve biochemical verification; however, large-scale population-based studies tend to show low levels of bias in self-reporting of smoking status (Benowitz et al., 2002), and we cannot identify any particular reasons in this study why participants would not have disclosed their true smoking status. Moreover, due to the relatively small sample size, especially at follow-ups, the study was not designed to assess continued abstinence. Although follow-up rates were relatively low, ‘missingness’ was not related to identity. There is a lack of reliable measures of nicotine dependence retrospectively, as for example the utility to predict a relapse based on the commonly used Heaviness of Smoking Index (Heatherton, Kozlowski, Frecker, Rickert, & Robinson, 1989) and its two components (cigarettes per day and time to first cigarette) declines rapidly from around the first month of a quit attempt (Yong et al., 2014). Therefore, we cannot rule out the possibility that participants' nicotine dependence prior to their most recent quit attempt was not accounted for sufficiently in the analysis. Even though using ‘cigarettes per day’ might not have been as good as other measures of nicotine dependence, such as urges to smoke (Fidler, Shahab, & West, 2010), we opted for this approach because we hypothesise that urges to smoke might partially mediate the association between identity and relapse, and this study did not aim to assess potential mediation effects. The measure used to assess identity might not have captured the potential richness of people's identities in relation to smoking after a quit attempt; however, in this first study we wanted to concentrate solely on the two major end-points of potential post-quit identities (i.e. smoker or non-smoker) to evaluate whether it merits further study. Finally, the extent to which participants' identification with categorical self-labels might have changed during the follow-ups was not assessed; therefore, the potential influence of subsequent changes in ex-smokers' identities over time on smoking abstinence could not be evaluated.

To our knowledge, this study is the first to provide evidence on the potential prospective associations between non-smoker identity following quitting and the lasting success of attempts to stop smoking in a representative sample of ex-smokers. A large proportion of smokers thought of themselves as non-smokers after having made a serious quit attempt in the past year, and people who have been abstinent for longer were more likely to do so than ex-smokers who quit more recently.

## Role of funding source

The Smoking Toolkit Study is partly funded by the English Department of Health, Pfizer, GlaxoSmithKline and Johnson and Johnson. Pfizer, GlaxoSmithKline and Johnson and Johnson are manufacturers of smoking cessation products who had no involvement in the design of the study, collection, analysis or interpretation of the data, the writing of the report, or the decision to submit the paper for publication.

## Contributors

Robert West designed the Smoking Toolkit Study. Ildiko Tombor managed the literature searches and summaries of previous related work, undertook the statistical analysis and wrote the first draft of the manuscript. All authors contributed significantly to the writing of subsequent versions and approved the final manuscript.

## Conflict of interest

Ildiko Tombor and Caitlin Notley do not have any conflict of interest to declare. Lion Shahab has received honoraria for talk and travel expenses from manufacturers of medications for smoking cessation to attend meetings and workshops. Jamie Brown has received an unrestricted research grant from Pfizer. Robert West has undertaken research and consultancy for companies that develop and manufacture smoking cessation medications.

## Figures and Tables

**Table 1:** Sample characteristics at baseline and at three month and six month follow-ups.

	Baseline			At three month follow-up		At six month follow-up	
	Total (*n* = 574)	Post-quit non-smoker identity (*n* = 465)	Post-quit smoker identity (*n* = 109)	Followed-up (*n* = 179)	Lost to follow-up (*n* = 395)	Followed-up (*n* = 163)	Lost to follow-up (*n* = 411)
Women, % (*n*)	56.1 (322)	55.1 (256)	60.6 (66)	59.8 (107)	54.4 (215)	55.8 (91)	56.2 (231)
Age, mean (SD)	41.5 (16.0)	40.7 (16.2)	45.3 (14.7)	45.9 (15.8)⁎	39.6 (15.8)	48.5 (15.7)⁎	38.8 (15.4)
C2DE social grade, % (*n*)	57.8 (332)	57.0 (265)	61.5 (67)	57.0 (102)	58.2 (230)	60.7 (99)	56.7 (233)
Number of cigarettes per day, mean (SD)	14.8 (10.0)	14.5 (9.7)	16.3 (10.9)	15.8 (10.7)	14.4 (9.6)	17.0 (11.5)⁎	14.0 (9.2)
Number of quit attempts in the past year at baseline, mean (SD)	1.4 (0.7)	1.3 (0.7)	1.5 (0.8)	1.3 (0.7)	1.4 (0.7)	1.3 (0.7)	1.4 (0.7)
Baseline length of abstinence, % (*n*)							
Less than a week	6.6 (38)	4.1 (19)	17.4 (19)	4.5 (8)	7.6 (30)	4.3 (7)	7.5 (31)
1–4 weeks	13.8 (79)	13.1 (61)	16.5 (18)	14.5 (26)	13.4 (53)	14.7 (24)	13.4 (55)
4–8 weeks	8.4 (48)	8.2 (38)	9.2 (10)	8.4 (15)	8.4 (33)	9.8 (16)	7.8 (32)
8–12 weeks	9.2 (53)	9.0 (42)	10.1 (11)	7.3 (13)	10.1 (40)	8.0 (13)	9.7 (40)
12–26 weeks	22.1 (127)	22.6 (105)	20.2 (22)	21.8 (39)	22.3 (88)	23.9 (39)	21.4 (88)
26–52 weeks	39.9 (229)	43.0 (200)	26.6 (29)	43.6 (78)	38.2 (151)	39.3 (64)	40.1 (165)
Unaided quit attempt, % (*n*)	57.8 (332)	60.6 (282)	45.9 (50)	56.4 (101)	58.5 (231)	49.7 (81)⁎	61.1 (251)
Non-smoker identity, % (*n*)	–	–	–	82.7 (148)	80.3 (317)	82.2 (134)	80.5 (331)

⁎ Significant differences (*p* < 0.05) between followed-up and lost to follow-up samples at three months and six months respectively.

**Table 2:** Associations between participants' socio-demographic and smoking-related characteristics and a non-smoker identity at baseline.

	Non-smoker identity vs. smoker identity at baseline (*n* = 574)	
	OR (95% CI); *p*-value	Adj. OR^a^ (95% CI); *p*-value
Women	0.80 (0.52–1.22); *p* = 0.299	0.77 (0.49–1.20) *p* = 0.245
Age (increase in 10 years of age)	0.84 (0.73–0.96); *p* = 0.008	0.84 (0.73–0.97); *p* = 0.017
C2DE social grade	0.83 (0.54–1.27); *p* = 0.394	1.00 (0.64–1.56); *p* = 0.982
Number of cigarettes per day (increase in 10 cigarettes per day)	0.84 (0.67–1.05); *p* = 0.132	0.96 (0.75–1.24); *p* = 0.762
Number of quit attempts in the past year	0.81 (0.62–1.05); *p* = 0.115	0.90 (0.68–1.19); *p* = 0.448
Baseline length of abstinence (increase by duration category)	1.32 (1.17–1.48); *p* < 0.001	1.31 (1.16–1.48); *p* < 0.001
Unaided quit attempt	1.82 (1.20–2.77); *p* = 0.005	1.54 (0.98–2.42); *p* = 0.064

a Adjusted for all study variables.

**Table 3:** Prospective associations between participants' baseline characteristics and self-reported smoking status at three month and six month follow-ups.

	Smoking abstinence vs. relapse at three month follow-up (*n* = 179)		Smoking abstinence vs. relapse at six month follow-up (*n* = 163)	
	OR (95% CI); *p*-value	Adj. OR^a^ (95% CI); *p*-value	OR (95% CI); *p*-value	Adj. OR^a^ (95% CI); *p*-value
Non-smoker identity	2.37 (1.07–5.21); *p* = 0.033	2.64 (1.09–6.42); *p* = 0.032	2.19 (0.97–4.97); *p* = 0.060	2.05 (0.83–5.04); *p* = 0.120
Women	0.46 (0.23–0.91); *p* = 0.025	0.49 (0.23–1.05); *p* = 0.067	0.57 (0.29–1.12); *p* = 0.106	0.69 (0.33–1.44); *p* = 0.326
Age (increase in 10 years of age)	1.11 (0.90–1.37); *p* = 0.325	0.99 (0.78–1.26); *p* = 0.930	1.18 (0.94–1.47); *p* = 0.155	1.13 (0.88–1.44); *p* = 0.335
C2DE social grade	0.95 (0.50–1.79); *p* = 0.866	1.10 (0.54–2.26); *p* = 0.791	0.87 (0.44–1.70); *p* = 0.682	0.97 (0.47–2.03); *p* = 0.944
Number of cigarettes per day (increase in 10 cigarettes per day)	1.39 (0.97–2.05); *p* = 0.074	1.22 (0.81–1.85); *p* = 0.338	1.20 (0.86–1.68); *p* = 0.288	1.06 (0.72–1.54); *p* = 0.777
Number of quit attempts in the past year	0.73 (0.49–1.09); *p* = 0.128	1.08 (0.65–1.80); *p* = 0.763	0.81 (0.52–1.25); *p* = 0.343	1.08 (0.63–1.84); *p* = 0.780
Baseline length of abstinence (increase by duration category)	1.58 (1.29–1.93); *p* < 0.001	1.58 (1.26–1.99); *p* < 0.001	1.43 (1.16–1.75); *p* = 0.001	1.41 (1.13–1.76); *p* = 0.003
Unaided quit attempt	0.60 (0.31–1.14); *p* = 0.119	0.54 (0.25–1.18); *p* = 0.123	0.98 (0.51–1.89); *p* = 0.956	0.86 (0.41–1.84); *p* = 0.703

a Adjusted for all study variables.
